# Beneficial Effect of Increased Tryptophan Intake on Its Metabolism and Mental State of the Elderly

**DOI:** 10.3390/nu15040847

**Published:** 2023-02-07

**Authors:** Cezary Chojnacki, Anita Gąsiorowska, Tomasz Popławski, Paulina Konrad, Marcin Chojnacki, Michal Fila, Janusz Blasiak

**Affiliations:** 1 Department of Clinical Nutrition and Gastroenterological Diagnostics, Medical University of Lodz, 90-647 Lodz, Poland; 2 Department of Gastroenterology, Medical University of Lodz, 92-213 Lodz, Poland; 3 Department of Pharmaceutical Microbiology and Biochemistry, Medical University of Lodz, 92-215 Lodz, Poland; 4 Department of Developmental Neurology and Epileptology, Polish Mother’s Memorial Hospital Research Institute, 93-338 Lodz, Poland; 5 Department of Molecular Genetics, Faculty of Biology and Environmental Protection, University of Lodz, 90-236 Lodz, Poland

**Keywords:** tryptophan, tryptophan metabolism, tryptophan metabolites, kynurenine pathway of tryptophan metabolism, the elderly, mood disorders, dietary intervention, food intake control

## Abstract

The elderly often suffer from sleep disorders and depression, which contribute to mood disorders. In our previous work, we showed that elderly individuals with mood disorders had a lower intake of TRP and recommended a TRP-based dietary intervention to improve the mental state of such individuals. In this work, we assessed the impact of a TRP-rich diet on the mental state of, and TRP metabolism in, elderly individuals with mood disorders. Forty elderly individuals with depression and sleep disorders and an equal number of elderly subjects without mood disorders were enrolled in this study. TRP intake was evaluated with the nutrition calculator. Patients with mood disorders had a lower TRP intake than their normal counterparts and received a TRP-rich diet with TRP content of 25 mg per kilogram of the body per day for 12 weeks. The mental state was assessed before and after this dietary intervention with the Hamilton Depression Rating Scale (HAM-D) and the Insomnia Severity Index (ISI). At those times, urinary levels of TRP and its metabolites 5-hydroxyindoleacetic acid (5-HIAA), L-kynurenine (KYN), kynurenic acid (KYNA), and quinolinic acid (QA) were determined by liquid chromatography with tandem mass spectrometry and related to creatinine level. After TRP-based dietary intervention, the score of ISI and HAM-D decreased by more than half. A correlation analysis reveals that TRP, 5-HIAA, and KYNA might have anti-depressive action, while KYN and QA—pro-depressive. The levels of TRP, 5-HIAA, and KYNA in urine of mood disorder patients increased, while the levels of KYN and QA decreased. In conclusion, dietary consumption of adequate amount of tryptophan has a beneficial effect on mental health of the elderly with mood disorders and improves metabolism of this amino acid. Therefore, a TRP-enriched diet may be considered as a component of the treatment of elderly individuals with mood disorders.

## 1. Introduction

L-tryptophan (TRP), an exogenous amino acid, is essential to produce many neurotransmitters and hormones [1,2,3]. Its metabolites exhibit oxidant/antioxidant, anti-inflammatory, neuroprotective, or neurotoxic activity [4]. In the human body, TRP undergoes metabolic transformation in three main pathways: serotonin (5-hydroxyptamine, 5-HT), kynurenine, and the microbiota-related indole pathway. In the serotonin pathway, TRP is converted to 5-hydoxytryptophan, and then to 5-hydroxytryptamine (serotonin) and to N-acetyl-5-metoxytryptamine (melatonin). The TRP metabolites produced in the serotonin pathway play an important role in the gastrointestinal tract and central nervous system (CNS) [5,6]. However, most, about 95%, of TRP is metabolized in the kynurenine pathway in which several TRP metabolites are produced, including L-kynurenine (KYN), quinolinic acid (QUIN), 5-hydroxyindoleacetic acid (5-HIAA), and kynurenic acid (KYNA) [7,8,9].

L-kynurenine along with its metabolites are important elements of the regulation of the central and peripheral nervous systems and, subsequently, they are significant players in the pathogenesis of neurological and psychiatric disorders [10]. L-kynurenine also regulates the immune response as it is an agonist of the aryl hydrocarbon receptor (AhR), a ligand-activated transcription factor regulating many physiological functions, including these involved in the control of the gut–brain axis [11,12]. Kynurenic acid affects brain functions as it is an antagonist for the 7 nicotinic acetylcholine receptors (7nAchRs) and the N-methyl-d-aspartic acid (NMDA) receptor [13]. It is also an agonist for G protein-coupled receptor 35 (GPR35) [14]. In addition, 3-hydroxykynurenine is generally neurotoxic as it can support oxidative stress induction and QUIN, as an NMDA receptor agonist, displays excitotoxic properties [15,16]. There has been a substantial increase in the research interest on the role of the KYN pathway of TRP metabolism in the nervous system and aging and we tried to link these two items in the present work.

According to WHO, major depressive disorder is a main reason of disability in the world [17]. However, the molecular mechanisms underlying this disease are unknown and many metabolic aspects are taken into consideration. These also include TRP metabolism in both serotonin and KYN pathways as well as inflammation [18]. The elderly are especially prone to depression due to chronic age-related diseases and cognitive impairment [19]. Also, metabolic processes can be severely affected in elderly individuals, with serious consequence for both physical and physic state [20].

Neurotoxic effect of kynurenines is fundamental for the serotonin–kynurenine hypothesis of depression, meaning that depression can be caused by a deficiency of serotonin or an excess of kynurenine metabolites [21,22,23]. For this reason, TRP supplementation has been used in the treatment of depression, although the results obtained so far are largely inconsistent [24]. Although TRP consumption even in high doses is generally considered as safe, it may increase the level of neurotoxic KYN metabolites [25]. Metabolism of TRP depends on many factors, including age and accompanying diseases. In our previous study, we found an decreased TRP intake in elderly individuals with mood disorders as compared with individuals without such disturbances [26]. Therefore, an increase in TRP content in the diet of elderly patients with mood disorders is rational.

In this work, we investigated TRP content in the diet of elderly individuals with or without mood disorders. Then, the former received a TRP-enriched diet for 12 weeks and their mental state was determined before and after such dietary intervention. Furthermore, the levels of the main metabolites of the KYN pathway of TRP metabolism were determined in the mood disorder group before and after the dietary intervention and such determinations were also made in individuals without mood disorders.

## 2. Materials and Methods

### 2.1. Patients

One hundred thirty-two individuals were recruited to this study in 2018–2022 in the Department of Clinical Nutrition and Gastroenterological Diagnostics and in the Department of Gastroenterology, Medical University of Lodz, Lodz, Poland. Initially, each patient was assessed for their mental condition using the modified Hamilton Depression Rating Scale (HAM-D) [27]. The following recommended criteria were adopted: a score of 0–7—no mental disorder; 8–12—mild depression; 13–18—moderate depression; 19–29—severe depression; over 30—very severe depression. The quality of sleep was estimated by the Insomnia Severity Index [28] with our own modification, replacing the evaluation of the quality of life (0–4 points) with the assessment of shortening of sleeping time. The score was interpreted as follows: 0–7 points—absence of insomnia; 8–14—threshold insomnia; 15–21—moderate insomnia; and 22–28—severe insomnia. Depression and sleep disturbances were further collectively referred as to mood disorders. The inclusion criteria for the mood disorders group were ISI score above 14 and HAM-D score above 15. The inclusion criteria for the no mood disorder group were ISI below 9 and HAM-D score below 8. A total of 80 subjects, 62 women and 18 men, aged 74–85 years, were selected for further study. They were divided into two groups, 40 individuals each, with or without mood disorders. 

Clinical tests were carried out to determine organic diseases. Mild hypertension (29/80–29.8%), stable coronary disease (15/80–18.7%), type 2 diabetes (19/80–23.75%), Hashimoto disease (11/80–13.25%), and gastrointestinal disorders (36–5.0%) were detected in some individuals. Exclusion criteria were very severe depression, circulatory or respiratory failure, advanced diabetes, liver diseases, renal failure, inflammatory bowel diseases, cancer, and taking psychotropic drugs or sleeping pills.

### 2.2. Laboratory Tests

Blood for testing was taken on empty stomach and the following routine tests were performed: blood cell count, C-reactive protein, count, glucose, bilirubin, urea, creatinine, profile of lipids, thyroid-stimulating hormone, free thyroxine, free triiodothyronine, vit. D3, vit. B12, alanine and asparagine aminotransferase, gamma-glutamyltranspeptidase, alkaline phosphatase, amylase, lipase, antibodies for tissue transglutaminase, and deaminated gliadin peptide. Fecal calprotectin (FC) was evaluated by sandwich ELISA test in Quantum Blue Reader (Buhlmann Diagnostics, Amherst, NH, USA). In addition, the ^13^C urea breath test was performed to exclude Helicobacter pylori infection and the lactulose hydrogen breath test was applied to eliminate patients with small intestinal bacterial overgrowth.

Urine samples for the analysis of TRP metabolites were collected in the morning on an empty stomach into a container with a solution of 0.1% hydrochloric acid as a stabilizer. L-tryptophan and its metabolites 5-HIAA, KYN, KYNA, and QA were determined in urine using liquid chromatography–tandem mass spectrometry (LC–MS/MS—Ganzimmun Diagnostics AG, Mainz, Germany; D-ML-13147–01-01). The levels of these metabolites were expressed in mg/g creatinine. The proportions between the levels of 5-HIAA acid TRP, as well as between KYN and TRP, were also calculated. The 5-HIAA/TRP and KYN/TRP ratios were considered as markers of the tryptophan hydrolase 1 and 2,3-dioxygenase activity, respectively.

### 2.3. Dietary Intervention

**Figure 1:**
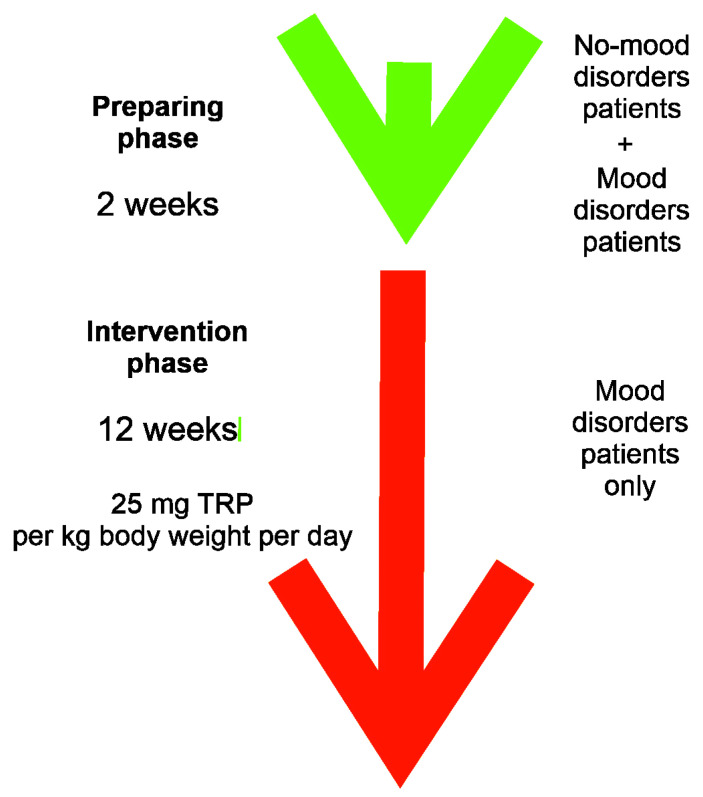
Schematic representation of the nature of dietary intervention. Two weeks before the beginning of the trial (preparation phase, green arrow) all (with and without mood disorders) patients started to monitor and daily record their diet without any changes in their eating habits. After that period, the intervention phase (red arrow) began, which lasted 12 weeks and in which the mood disorder patients only were enrolled. They were instructed to change their diet according to a dietician’s recommendation. New diet contained 25 mg of tryptophan (TRP) per kg of body weight per day.

All individuals were recommended to record in the nutritional diary the type and quantity of products consumed every day for 14 days prior to investigations (preparing phase, Figure 1). The average daily TRP intake was calculated using the nutritional calculator with the application Kcalmar.pro-Premium (Hermex, Lublin, Poland). The patients applied the balanced diet of total caloric value 2000 kcal and with daily intake a minimum of 50 g of proteins, 270 g carbohydrates, and 70 g fats. On the day of the evaluation, everyone was administrated the diet with the TRP content calculated in advance. The content of TRP in food products was determined in accordance with findings of the Polish National Institute of Public Health, Warsaw, Poland. Products with a rich TRP content, including wheat bread, sweets, hard cheeses, meat and some fish, as well as raw fruit and vegetables, were included in the diet, and the optimal amount of proteins, carbohydrates fats, and vitamins was maintained.

Subsequently, after educational instructions, the patients with mood disorders were commanded to include to their diet 25 mg TRP per kilogram of body weight per day for 12 weeks (intervention phase, Figure 1). These patients were also recommended to complete a diet diary daily, under the control of nutritionists, whom they contacted by phone or e-mail. The recommended diet was varied individually, considering the accompanying diseases and eating habits. Meals were prepared at home. Patients also recorded the medicaments taken and their doses. After each week, the amount of TRP intake was analyzed to evaluate compliance with the recommendations and introduce necessary changes. Follow-up medical examinations with the assessment of the somatic and mood symptoms and laboratory tests were performed after 12 weeks.

### 2.4. Ethical Issues

This study was conducted in accordance with the Declaration of Helsinki and the principles of Good Clinical Practice. Written consent was obtained from each subject enrolled in the study and the study protocol was approved by the Bioethics Committee of Medical University of Lodz (RNN/176/18/KE). The study was designed and conducted as an open-label clinical trial.

### 2.5. Data Analysis

Normality of the data distribution was checked by Shapiro–Wilk’s W test. The *t*-Student’s test and Mann–Whitney U test were used to compare differences between two groups. The correlations between the quantitative variables were analyzed using the Spearman’s rank test. Differences within groups before and after dietary intervention were analyzed using the Wilcoxon signed-rank test. All statistical analyzes were performed with STATISTICA 13.3 software (TIBCO Software INC., Palo Alto, CA, USA). metabolism.

## 3. Results

There are no differences in routine laboratory tests results between individuals with and without mood disorders before the dietary intervention (Table 1). Both groups differ significantly in sleep quality, evaluated by the ISI score and depressive behavior assessed by the HAM-D scores. These differences are definite as the values of the scores range 2–3 times. Apart from differences in ISI and HAM-D scores, individuals with mood disorders consume about 36% less TRP than subjects without such disturbances (*p* < 0.001). However, that difference is more pronounced when the amount of consumed TRP is related to the body weight, by over 40% (*p* < 0.001).

**Figure 2:**
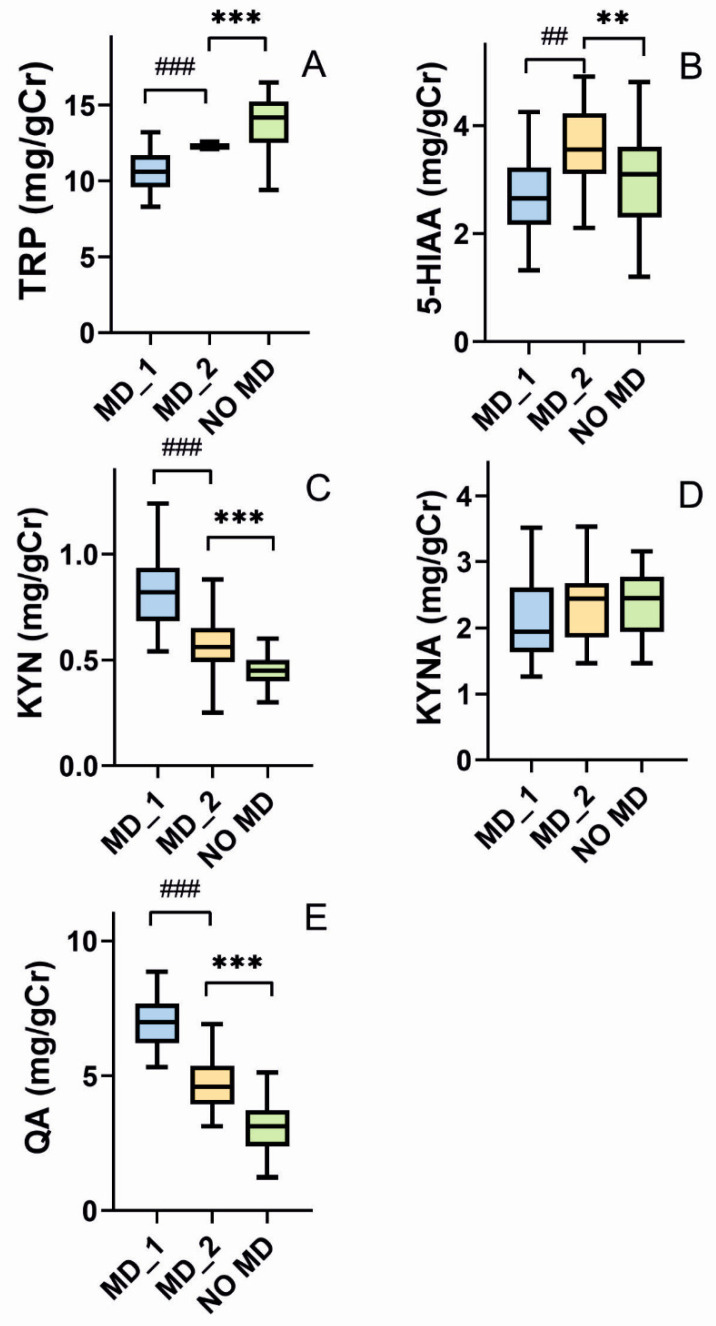
Urinary levels of tryptophan (TRP) (**A**), 5-hydroxyaminoacetic acid (5-HIAA) (**B**), kynurenine (KYN) (**C**), kynurenic acid (KYNA) (**D**), and quinolinic acid (QA) (**E**) expressed in milligram per gram of creatinine (mg/g Cr), in elderly individuals with mood disorders before (MD_1) and after (MD_2) a 12 week dietary intervention with TRP as well as elderly subjects without mood disorders (NO MD). Median with boxes represent I and III quartiles, and error bars represent 1.5 times the interquartile distance. The Wilcoxon matched-pairs signed-rank test was used to compare before–after groups (denoted as #), otherwise U Mann–Whitney test was used to analyze differences within two groups (denoted as *); *n* = 40 in NO MD and *n* = 38 in MD-1 and MD-2; **—*p* < 0.01; ***—*p* < 0.001; ##—*p* < 0.01, ###—*p* < 0.001.

Dietary intervention with TRP-rich products increases urinary levels of TRP (Figure 2A, *p* < 0.001) and 5-HIAA (Figure 2B, *p* < 0.001) in mood disorders individuals. The levels of TRP and its metabolites in patients with mood disorders after dietary intervention are higher than corresponding levels in subjects without mood disorders (*p* < 0.01). The mood disorders group have a lower median concentration of KYN after dietary intervention (*p* < 0.001, Figure 2C), but it is still higher than that observed in the no mood disorders group (*p* < 0.001). Increased intake of TRP does not change KYNA concentration in the mood disorders group, which is at the same level as in individuals without mood disorders (Figure 2D, *p* > 0.05). The course of changes in the QA levels (Figure 2E) are the same as KYN.

**Figure 3:**
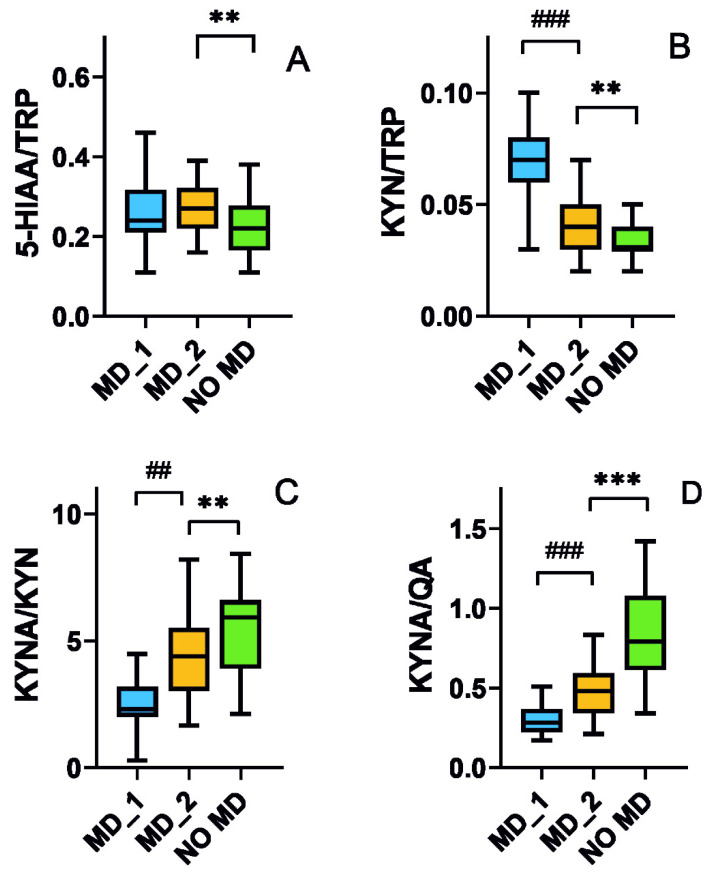
Ratios of urinary levels of 5-hydroxyaminoacetic acid (5-HIAA) (**A**) and kynurenine (KYN) (**B**) to tryptophan (TRP), as well as kynurenic acid (KYNA) to KYN (**C**) and quinolinic acid (QA) (**D**) expressed in milligram per gram of creatinine (mg/g Cr), in elderly individuals with mood disorders before (MD_1) and after (MD_2) a 12 week dietary intervention with tryptophan as well as elderly subjects without mood disorders (NO MD). Median with boxes represent I and III quartiles, and error bars represent 1.5 times the interquartile distance. The Wilcoxon matched-pairs signed-rank test was used to compare before–after groups (denoted as #), otherwise U Mann–Whitney test was used to analyze differences within two groups (denoted as *); *n* = 38 in the MD groups and *n* = 40 in NO MD group; **—*p* < 0.01; ***—*p* < 0.001; ##—*p* < 0.01, ###—*p* < 0.001.

We do not observe any effect of the dietary intervention on the 5-HIAA/TRP ratio, but after the intervention, the ratio is still higher than in the no mood disorders group (*p* < 0.01, Figure 3A). Patients with mood disorders display a lower KYN/TRP ratio after dietary intervention (*p* < 0.01, Figure 3B), but it is still higher than in the no mood disorders group (*p* < 0.01). Mood disorders patients display increased KYNA/KYN ratio (*p* < 0.01), but it is still lower than in the no mood disorders group (*p* < 0.01, Figure 3C). Dietary intervention increases the KYN/QA ratio (*p* < 0.001), but it is still lower than in patients without mood disorders (*p* < 0.001, Figure 3D).

**Figure 4:**
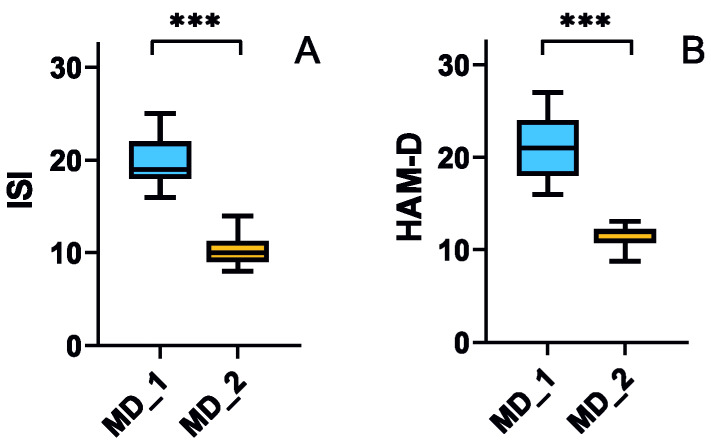
Effect of a 12-week dietary intervention with tryptophan on the sleep disturbances measured with Insomnia Severity Index (ISI, (**A**)) and level of depression measured with Hamilton Depression Rating Scale (HAM-D, (**B**)) in elderly individuals with mood disorders before (MD_1) and after (MD_2) the intervention. Median with boxes representing I and III quartiles, error bars represent 1.5 times the interquartile distance. The Wilcoxon matched-pairs signed-rank test was used to compare before–after groups; *n* = 40 for MD_1 and *n* = 38 for MD_2; ***—*p* < 0.001.

Figure 3 presents the effect of the dietary intervention on the mental state of the mood disorder patients. The 12 week diet with increased content of TRP significantly lowers both ISI and HAM-D scores in patients initially classified as individuals with mood disorders (*p* < 0.001 for both ISI and HAM-D, Figure 4A and Figure 4B, respectively). It is worth noting that after the intervention, the median ISI and HAM-D scores are below the inclusion criteria for the mood disorder group, but still higher than such criteria for the no mood disorder group.

In the next step, we looked for the possible correlation between HAM-D and levels of TRP and its metabolites.

Severity of depressive symptoms is positively correlated with KYN and QA levels and negatively correlated with TRP, 5-HIAA, KYNA, and the KYNA/KYN and KYNA/QA ratios in subjects with mood disorders (Table 2). No other statistically significant correlations are observed. Therefore, we assume that in our conditions, patients, and methods, KYN and QA are neurotoxic while TRP, 5-HIAA, and KYNA might exert a protective, anti-depressive effect. No correlations are found between ISI scores and the levels of TRP, its metabolites in the KYN pathway, and the ratios of their concentrations.

## 4. Discussion

In this work, we show that a TRP-enriched diet improves the mental state and tryptophan metabolism in an elderly population. Elderly individuals with mood disorders have a lower TRP intake with their diets than age-matched subjects without mental complications (Table 1), but diet-enrichment with 25 mg of TRP/day for 12 weeks results in an amelioration of depressive behavior and sleep disorders (Figure 4). Moreover, a TRP-enriched diet reduces the level of neurotoxic agents and increases the concentration of agents beneficial for the nervous system in urine (Figure 2 and Figure 3).

We performed our research in an open-label trial mode without a placebo group, with all participants receiving the same treatment. It would be very difficult, if not impossible at all, to run our trial in a placebo-controlled mode, as our treatment was based on the changes in diet composition, not diet supplementation. Moreover, the placebo-controlled mode would decrease the statistical power of our results as the available patient group would have been divided into two smaller subgroups.

Our dietary intervention did not change dietary habits of mood disorder individuals but changed proportions of specific foods in their everyday diet. It is tempting to ask whether some kinds of foods consumed by the patients with mood disorders supported improvement in their mental state. We are not able to answer this question, as there was not any representative diet before and after the intervention. That group of elderly patients, suffered from various disorders, enforced an elimination diet in many cases. One example is diabetes mellitus, which featured in some of the patients, who had to adjust their diet to physicians’ recommendations. The group of patients with no mood disorder had an adequate level of TRP and, therefore, a TRP-enriched dietary intervention was not justified within this research project. Therefore, those patients only served as a type of control and were not included in subsequent experiments after initial determinations.

The obtained results confirm our earlier findings showing a low TRP intake in mood disorders patients [18]. These results are in line with results obtained elsewhere [29,30]. However, some outcomes of the relationship between TRP intake and mental state are controversial. Acute TRP depletion influenced slightly the mental state of normal [31,32,33], and depressed individuals [34,35,36]. Other studies showed that the diet with higher TRP content resulted in a decrease in depressive symptoms and anxiety [37,38,39,40]. Administration of TRP increases its brain concentration and stimulates the synthesis of serotonin and melatonin, which improve mood and sleep quality [41,42]. Moreover, the role of nutrition in the onset and progression of mental diseases is controversial. In some clinical trials, TRP was administrated in high doses, significantly exceeding physiological requirement for this amino acid [24,43]. High doses of tryptophan may cause harmful side effects and change not only serotonin synthesis, but also components of the KYN pathway [44,45]. Changes in TRP metabolic pathways can be influenced by many factors, including gut microbiota and aging processes [46,47]. Serotonin neurons and neurotransmitters are impaired in aging, and their dysfunction may predispose a person to developing depressive symptoms and sleep disturbances [48,49,50]. Pharmacological treatment of these disorders in the elderly is challenging due to potential side effects. A balanced, personalized diet is fundamental for a good somatic and mood state in all patients [51,52]. Nutrition is especially important in the elderly due to changes in health and social status [53]. Generally, it is recommended to increase consumption of fruits, vegetables, legumes, whole grain cereals, nuts, seeds, and foods rich in polyunsaturated fatty acid and vitamins, while reducing consumption of total fat, processed foods, sugars, and sweets [54,55,56]. Results of several studies suggest a beneficial effect of a TRP-rich diet in individuals with depressed mood. An average daily intake of TRP is 3.5–5.0 mg per kg body weight (250–425 mg in total), and this level of TRP is needed to keep the nitrogen balance in the human body [57].

In our study, daily consumption of TRP in elderly individuals with mood disorders was lower than in age-matched subjects without mood disorders (Table 1). We observe significant changes in TRP metabolism in mood disorder patients—the levels of KYN, QA, and the KYN/TRP ratio are higher than in the no mood disorders group (Figure 1 and Figure 2). Higher QA levels are reported in patients with depression, with concomitant decreased level of KYN and KYNA [24,45]. Other studies show a prevalence of neurotoxic TRP metabolites in depression, especially occurring in late-life [58,59,60]. The reasons for these differences are unclear, but most of these studies include patients at different ages and treated with various antidepressants in varying doses. In general, there is not any general consensus on the toxic or protective nature of products of TRP metabolism. There are not standards for the dietary treatment with, and determination of, these products either.

We observe some important correlations between severity of depression and concentrations of TRP metabolites in the KYN pathway. We consider them as important as they may reflect conditions of our patients and trial. KYN and QA are pro-depressive, while TRP, 5-HIAA, and KYNA might exert protective, anti-depressive effects (Table 2). However, it should be noted that the correlations presented in Table 2 provide only more or less likely association without pointing at any causal relationship, and we would like to underline that these correlations are specific for our research design and our patients. Moreover, these correlations are mostly weak (ρ 0.23–0.57). This is important considering heterogeneity of patients and treatment in several other studies. 

In the light of the categorization of TRP, 5-HIAA, and KYNA as anti-depressive and KYN and QA as pro-depressive, the 12 week dietary intervention with TRP results in an improvement in TRP metabolism in elderly individuals with mood disorders, as we observe an increased level of TRP but decreased levels KYN and QA after intervention (Figure 1). Moreover, the KYNA/KYN and KYNA/QA ratios increase, but the KYN/TRP ratio decreases after the intervention. However, the levels of all metabolites and their ratios do not reach the levels typical for patients without mood disorders. The immediate reason for this may be too low a content of TRP in the intervention diet. This may suggest a perspective of increasing the content of tryptophan in further studies. However, we determined only two aspects of the mental state of the patients, evaluated by the HAM-D and ISI scores. In fact, a more complete evaluation would be more reliable and informative. In particular, cognitive functions might be evaluated, as they are strongly related to age and associated with depressive behavior.

The main result obtained in our study is presented in Figure 3—dietary intervention with increased TRP content decreases markers of mood disorders in elderly patients. Again, lowered HAM-D and ISI score medians are still above medians for individuals without mood disorders.

Apart from an incomplete assessment of the mental state of the patients, our study has another limitation, as we investigated only one pathway of TRP metabolism—the kynurenine pathway. Although this pathway is responsible for metabolism of about 95% of TRP, the other pathway, the serotonin pathway, should be also considered. We can only speculate that the decrease in toxic TRP metabolites, KYN, and QA was associated with an increased activity of serotonin pathway and this should be explored in further research. 

In summary, 12 week dietary intervention with increased tryptophan content in elderly individuals with mood disorders evaluated by Hamilton Depressive Rating Scale and Insomnia Severity Index resulted in the improvement of tryptophan metabolism in that sense that an increased amount of neuroprotective and decreased amount of neurotoxic metabolites were produced. This intervention decreased the values of both scales to the values only slightly higher than those typical for individuals without such mental problems. 

## 5. Conclusions

Dietary tryptophan may ameliorate moderate depressive behavior and sleep disturbances in elderly adults. Further studies are needed to determine what components of the kynurenine pathway of tryptophan metabolism are directly responsible for these beneficial effects to project a more specific and efficient dietary intervention in the elderly to improve their mental state.

## Figures and Tables

**Table 1:** Characteristics of individuals without and with mood disorders enrolled in this study; *n* = 40 in either group.

Feature *^a^*	No Mood Disorders	Mood Disorders
Age (years)	74.9 ± 7.3	76.2 ± 8.6
Gender (M/F)	18/22	14/26
BMI (kg/m^2^)	23.8 ± 1.9	22.4 ± 2.3
CRP (mg/L)	1.83 ± 0.33	3.64 ± 2.83
AST (U/L)	16.4 ± 3.7	19.1 ± 6.2
ALT (U/L)	16.1 ± 5.2	21.2 ± 8.9
TSH (µIU/mL/L)	2.07 ± 0.92	1.68 ± 1.22
HbA1c (mmol/mol)	36.3 ± 3.4	1.68 ± 1.22
Creatinine (mg/dL)	0.75 ± 0.23	0.86 ± 0.21
GFR (mL/min)	98.6 ± 10.7	89.1 ± 10.3
ISI score	9.6 ± 1.19	19.7 ± 6.2 ***
HAM-D score	7.01 ± 1.12	20.8 ± 4.26 ***
TRP (mg daily)	1256 ± 193	806 ± 174 ***
TRP (mg/body weight daily)	21.2 ± 4.23	12.5 ± 3.96 ***

*^a^* average ± SD (standard deviation), BMI—body mass index, CRP—C-reactive protein, FC—faecal calprotectin, AST—aspartate aminotransferase, ALT—alanine aminotransferase, TSH—thyroid-stimulating hormone, HbA1c—glycated hemoglobin, GFR—glomerular filtration rate; ISI—Insomnia Severity Index; HAM-D—Hamilton Depression Rating Scale; TRP—tryptophan intake; differences between groups were assessed by Student’s *t*-test, ***—*p* < 0.001.

**Table 2:** Correlation between the Hamilton Depression Rating Scale (HAM-D) score and levels of tryptophan (TRP) and its metabolites in urine of elderly individuals with mood disorders. The correlations were analyzed using the Spearman rank test.

Pair of Variables *^a^*	Spearman’s ρ	*p*
HAM-D and TRP	−0.574738	<0.001
HAM-D and 5-HIAA	−0.450369	<0.001
HAM-D and KYN	0.537382	<0.001
HAM-D and KYNA	−0.229663	<0.05
HAM-D and QA	0.739648	<0.001
HAM-D and KYNA/KYN	−0.484408	<0.001
HAM-D and KYNA/QA	−0.540872	<0.001

*^a^* 5-HIAA—5-hydroxyindoleacetic acid, KYN—kynurenine, KYNA—kynurenic acid, QA—quinolinic acid; *n* = 40, ρ—Spearman’s rank correlation coefficient.

## Data Availability

Not applicable.
